# Dr. Milton Colt Belknap

**Published:** 1913-07

**Authors:** 


### OBITUARIES.

*Readers are requested to report promptly the death of any physician in Western New York, or former residents of this region, or graduates of any medical school in Western New York and to call the attention of the families of the deceased, our desire to publish adequate obituary notices.*

Dr. Milton Colt Belknap, New York Ecclectic 1868, died at his home after a lingering illness, May 22. He was born in Arcade in 1842. While a student in the Michigan Agricultural College in 1862, he enlisted in the 23d Michigan Infantry and served through the remainder of the Civil War. He practiced in Sinclairville and Brocton before coming to Mayville 21 years ago. He was a prominent Mason.

